# Proinflammatory and cardiovascular biomarkers are associated with echocardiographic abnormalities in children with HIV taking antiretroviral therapy

**DOI:** 10.1097/QAD.0000000000003368

**Published:** 2022-08-23

**Authors:** Edith D Majonga, Louis-Marie Yindom, Dan Hameiri-Bowen, Justin Mayini, Andrea M Rehman, Juan P Kaski, Hilda A Mujuru, Sarah L Rowland-Jones, Rashida A Ferrand

**Affiliations:** 1 Biomedical Research and Training Institute, Harare, Zimbabwe; 2 Department of Medical Physics and Imaging Sciences, Faculty of Medicine and Health Sciences, University of Zimbabwe, Harare Zimbabwe; 3 University of Oxford, Nuffield Department of Medicine, Oxford, United Kingdom; 4 MRC International statistics and epidemiology group, London School of Hygiene and Tropical Medicine, London, United Kingdom; 5 University College London Institute of Cardiovascular Science, London, United Kingdom; Centre for Inherited Cardiovascular Diseases, Great Ormond Street Hospital, London, United Kingdom; 6 Child and Adolescent Health Unit, Faculty of Medicine and Health Sciences, University of Zimbabwe, Harare, Zimbabwe; 7 Department of Clinical Research, London School of Hygiene & Tropical Medicine, London, United Kingdom

**Keywords:** biomarkers, perinatal HIV, ART, children, echocardiographic abnormalities

## Abstract

**Objectives:**

Children with perinatally-acquired HIV (PHIV) and taking antiretroviral therapy (ART) have a high prevalence of subclinical cardiac disease. We hypothesised that cardiac disease may be a consequence of dysregulated systemic immune activation driven by HIV infection. We examined cardiovascular and proinflammatory biomarkers and their association with echocardiographic abnormalities in children with PHIV.

**Design:**

Cross-sectional analysis of soluble biomarkers from a prospective cohort of children aged 6-16 years with PHIV and age-matched HIV-uninfected comparison group.

**Methods:**

Cryopreserved plasma samples were used to measure seven soluble biomarkers using multiplex bead assay (Luminex). Multivariable logistic regression assessed how biomarker levels related to cardiac abnormalities.

**Results:**

A total of 406 children participated in this study (195 PHIV and 211 HIV-uninfected). Mean (standard deviation (SD)) ages of PHIV and HIV-uninfected participants were 10.7 (2.6) and 10.8 (2.8) years, respectively. Plasma levels of CRP, TNF-α, ST2, VCAM-1 and GDF-15 were significantly higher in the PHIV group compared to uninfected control (p<0.001). Among children with PHIV, with one-unit representing one SD in biomarker level, a one-unit increase in CRP and GDF-15, was associated with increased odds of having left ventricular (LV) diastolic dysfunction [adjusted odds ratio (aOR), 1.49 (1.02–2.18; P<0.040)] and [aOR 1.71 (1.18–2.53; P=0.006)] respectively. Each one unit increase in GDF-15 was associated with increased odds of LV hypertrophy [aOR 1.84 (95% CI 1.10-3.10; p<0.021)].

**Conclusion:**

Children with PHIV had higher levels of proinflammatory and cardiovascular biomarkers compared to HIV-uninfected children. Increased CRP and GDF-15 were associated with cardiac abnormalities in children with PHIV.

## Introduction

Prior to widespread adoption of antiretroviral therapy (ART), cardiac disease in children was an indicator of advanced HIV infection and typically manifested as dilated cardiomyopathy and congestive heart failure [1–4]. This has become rare since the scale up of ART. However, several studies in the ART era report a high burden of subclinical cardiac disease, typified by left ventricular (LV) diastolic dysfunction [5, 6], LV hypertrophy and RV dilatation [7–10].

The underlying mechanism of cardiac disease in individuals with HIV is poorly understood. HIV infection is associated with dysregulated systemic immune activation, and in adults several studies have shown an association of non-AIDS defining illnesses, including cardiovascular disease, with markers of chronic inflammation [11]. Pro-inflammatory biomarkers including interleukin 6 (IL-6), tumour necrosis factor-alpha (TNF-α) and C-reactive protein (CRP) are reportedly elevated in adults with HIV and associated with several cardiovascular conditions [12].

Cardiac biomarkers, including N-terminal pro B-type Natriuretic Peptide (NT-proBNP) and cardiac troponin I (cTnI), are released in response to cardiac damage and stress and have a major diagnostic and prognostic role across a wide spectrum of cardiac diseases [13–18]. More recently, growth differentiating factor-15 (GDF-15) and suppression of tumorigenicity 2 (ST2) have been found to predict cardiovascular events in both the general population and in adults with HIV [14, 19, 20]. These biomarkers are released by cardiomyocytes and upregulated in response to mechanical strain [14]. Adhesion molecules including intercellular and vascular adhesion molecules 1 (ICAM-1 and (VCAM-1, respectively) are associated with endothelial dysfunction and strongly linked to a higher risk of cardiovascular disease in the general population [21].

Children with PHIV have higher markers of immune activation and inflammation compared to HIV-exposed but uninfected controls [13] and more recently, these markers were found to be associated with HIV-related comorbidity, chronic lung disease in PHIV [22]. However, little is known about the effect of HIV on specific cardiovascular and pro-inflammatory biomarkers and their association with cardiac abnormalities among children in the contemporary ART era. The main objectives of this study were to examine cardiovascular and proinflammatory biomarkers and to determine their association with cardiac structural and functional abnormalities in children with PHIV.

## Methods

### Study design

The INHALE (Investigation of heart and lung disease) was a prospective cohort study, conducted between August 2014 and December 2017, that investigated heart and lung diseases in older children and adolescents aged between 6-16 years, on ART for at least 6 months with no acute clinical symptoms.

The study enrolled 201 children with PHIV and 282 HIV-uninfected controls. Children with PHIV were followed up at 18 months. Details of the inclusion/exclusion criteria as well as findings on lung and cardiac diseases of INHALE study have been published elsewhere [7, 23–26]. Ethical approval was obtained from the Medical Research Council of Zimbabwe, Biomedical Research and Training Institute (BRTI) and London School of Hygiene & Tropical Medicine.

Transthoracic echocardiograms were performed at baseline and 18-month follow-up in children with PHIV only [7, 23], according to American Society of Echocardiography (ASE) recommendations [27]. Briefly, the following cardiac measures were performed: left ventricular (LV), right ventricular (RV), left (LA) and right atrial (RA) dimensions and function. Cardiac dimensions were converted to z-scores using local references [25], to provide LV and RV diameter in diastole z-scores (LVddz) and (RVddz) respectively, interventricular septum in diastole z-scores (IVSdz), LV posterior wall in diastole z-scores (LVPWdz), LA and RA z-scores and finally tricuspid annular plane systolic excursion (TAPSE) z-scores.

### Measurement of soluble biomarkers

Cryopreserved baseline plasma samples from the INHALE study participants were used to investigate the levels of circulating biomarkers. Seven biomarkers associated with cardiovascular disease in individuals without HIV were selected. Plasma levels of the following soluble biomarkers: CRP, GDF-15, ICAM-1, IL-6, ST2, TNF-α and VCAM-1 were measured using the Luminex multiplex bead assay on a MagPix instrument. On the day of the assay, cryopreserved plasma samples were thawed and equilibrated to room temperature. Samples were then spun at 16,000 xg for 4 minutes immediately prior to dilution. Assays were then run as per manufacturer’s protocol (Luminex technology, Hertogenbosch, Netherlands). Each sample and the standards used to create each standard curve were run in duplicate. The means of the technical replicates were taken, and the values from the blank fluorescence intensity were subtracted from each reading. Standard curves for each analyte were plotted using five parameter logistic curve fitting. Where samples were diluted, the concentration read from the standard curve were multiplied by the dilution factor to generate a final reading. Where samples had measurements falling outside the standard curve, they were repeated at an appropriate dilution. After repeats, biomarkers with detectable levels falling below the standard curve were assigned half the lower limit of quantification. All panels were run at the Biomedical Research and Training Institute laboratory in Harare.

### Statistical Analysis

Data were analysed using STATA 15 (StataCorp, Texas, USA) and R Studio (Version 1.1.383). Baseline demographic and clinical characteristics from the INHALE study are presented as means (standard deviation (SD)) or median ± interquartile ranges (IQR) or proportions with corresponding percentages (*n*, %), as appropriate. Comparison of clinical characteristics between participants with and without HIV was done using chi-squared or independent t-tests, as appropriate. Differences in biomarker levels between groups, either those with and without HIV or (among those with HIV) those virally suppressed (HIV viral load <400 copies/ml) or not were assessed using Wilcoxon rank sum test. Comparisons of biomarker levels between virally suppressed children with PHIV and those without HIV were done as a sensitivity analysis.

A correlation matrix between the biomarkers under study was constructed using the ggcorplot package in R. Spearman rank correlation coefficients were calculated for each pair of biomarkers in the HIV-positive and HIV-negative group. We explored relationships between biomarkers and associations between biomarkers and echocardiographic Z-scores (for RV and LV diameter and wall thickness, RV function and LA diameter) for those with HIV. Biomarker levels were first transformed into standardised log_10_ levels. The association between log_10_-transformed biomarkers and echocardiographic Z-scores was explored using linear regression in univariable and multivariable analysis adjusting for continuous age, sex, body surface area and log_10_ viral load. Multivariable logistic regression was used to determine the odds of having specific cardiac abnormalities, including LV diastolic dysfunction, LV hypertrophy, RV and LA dilatation in relation to continuous transformed biomarker levels, with a one-unit change in biomarker signifying a one standard deviation increase. A p-value of 0.05 or less was considered significant.

## Results

A total of 406 participants with available stored plasma samples were included in the analysis (n=195 with HIV, 98 (48%) female and n=211 without HIV, 109 (52%) female). Those excluded did not have stored baseline plasma samples (n= 77). Mean ages were similar in HIV and HIV-uninfected participants. Participants with HIV were more likely to be stunted (p<0.001). Among participants with HIV, median (IQR) age at ART initiation was 6 (3 – 8) years and duration on ART was 4.8 (2.7 – 6.4) years; 152 (78%) were virally suppressed (Table 1).

Echocardiographic data were available for 191/195 (98%) PHIV at baseline. Of these, 80 (42%) had cardiac abnormalities (LV dilatation 8 (4%); LV hypertrophy 21 (11%); LV diastolic dysfunction 43 (23%) and systolic dysfunction 3 (2%); LA dilatation 16 (8%); dilated cardiomyopathy 1(1%); RV dilatation 12 (6%) and systolic dysfunction 4 (2%) [7].

### Comparison of biomarkers between children with PHIV and children without HIV

**Figure 1:**
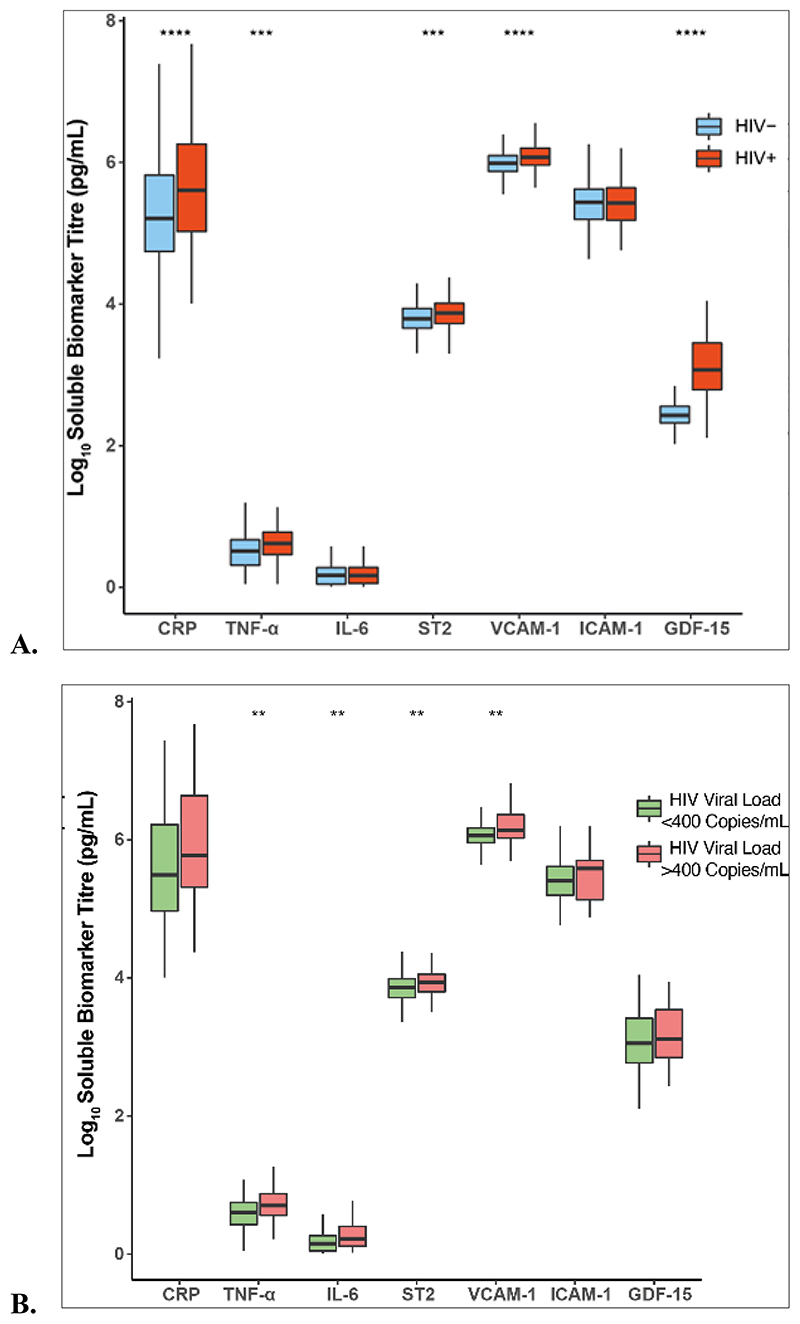
Comparison of biomarkers by HIV status and Viral suppression A) shows the comparison of log_10_ biomarker levels between HIV+ and HIV- groups and B) is a comparison of log_10_ biomarker levels by viral suppression within the HIV+ group using Wilcoxon rank sum test. Stars represent levels of significance, * 0.05, ** 0.01, ***0.001, **** 0.0001

The distribution of circulating biomarkers by HIV status and viral suppression is shown in Figure 1. Inflammatory biomarker levels including CRP and TNF-α, were higher among PHIV compared to uninfected controls, (p<0.001). Similarly, levels of GDF-15, VCAM-1 and ST2 were elevated in PHIV participants compared to children without HIV (p<0.001). There was no difference in IL-6 and ICAM-1 levels by HIV status. Among participants with HIV, those who were virally suppressed had lower TNF-α, IL-6, ST2 and VCAM-1 (p=0.01). Virally suppressed children had higher levels of CRP, GDF-15, and VCAM-1 (p<0.001), TNF-α and ST2 (p<0.01) compared to uninfected controls.

### Relationship between biomarkers and echocardiographic findings in participants with PHIV

**Figure 2:**
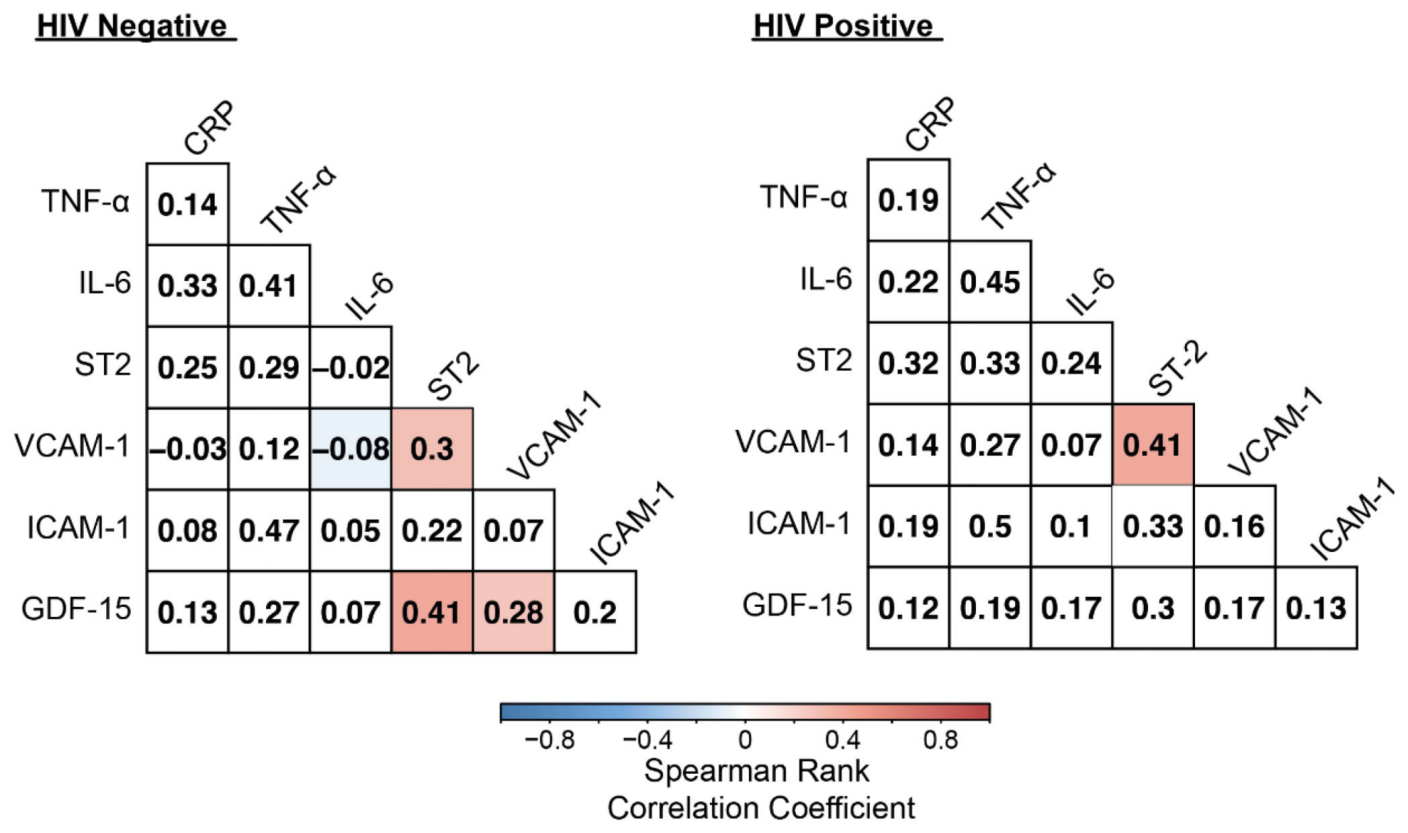
Spearman rank correlations between biomarkers among those without and with HIV Number within square indicates Spearman rank correlation coefficient between biomarkers. Correlations are significant at (p <0.05).

Biomarkers were moderately correlated for ST2 and VCAM-1 in PHIV while GDF-15, VCAM-1 and ST2 were correlated in children without HIV (p<0.05) (Figure 2). GDF-15 and VCAM-1 were significantly associated with higher interventricular septum z-scores (IVSdz) (adjusted β, 0.317 ; p<0.001) and (0.177 ; p=0.029), respectively. GDF-15 and IL-6 were associated with increased LV posterior wall z-scores (LVPWdz), while only GDF-15 was associated with lower RV diameter z-scores (RVDdz) (Table 2).

A one-unit increase in CRP and GDF-15 was associated with increased odds of having a cardiac abnormality increased, 1.50 (95% confidence interval (CI) 1.10 – 2.03; p=0.010)and 1.53 (95% CI 1.03– 2.27; p=0.035) respectively, after adjusting for age category, sex, body surface area and viral suppression (Table 3). Each one-unit increase in CRP and GDF-15 was associated with increased odds of LV diastolic dysfunction, 1.49 (95% CI 1.02–2.18; P=0.040) and 1.71 (95% CI 1.17–2.52; p=0.006), with the odds of LV hypertrophy increased by 1.84 (95% CI 1.10–3.10; p<0.021) for a one-unit change in GDF-15. There was an associated decreased risk of LV diastolic dysfunction with every one unit increase of ICAM-1, 0.73 (95% CI 0.56 – 0.96; p=0.023)

## Discussion

In this study, we explored cardiovascular and proinflammatory biomarkers and their association with echocardiographic measures of structure and function in children with PHIV. Overall, children with PHIV had elevated levels of CRP and TNF-α compared to those without HIV and similarly these biomarkers were also higher in virally suppressed children compared to uninfected controls, suggesting persistent systemic inflammation despite ART. CRP was associated with having a cardiac abnormality and, more specifically, LV diastolic dysfunction. Signs of inflammation have been previously observed in the myocardium of adults with HIV using cardiac magnetic resonance imaging [28]. Our findings support the hypothesis that systemic inflammation may play a role in the pathogenesis of HIV-related cardiac disease. While ART reduces immune activation, it does not completely normalise, and levels of proinflammatory biomarkers may remain elevated among those with HIV [29]. Chronic inflammation may cause cardiomyopathy through promoting apoptosis, fibrosis and inducing hypertrophy through alterations in the extracellular matrix [30, 31]. In particular, TNF-α exerts negative inotropic effects which result in contractile dysfunction [32]. Therefore, it is plausible that persistent inflammation and suboptimal immune recovery underlie the altered cardiac structure and function observed in children with PHIV.

Circulating biomarkers are dynamic and may be influenced by the complex interaction between metabolic, inflammation and viral load status in HIV. Notably, we found that viral suppression was associated with reduced TNF-α and IL-6. It is likely that in this cohort of children with PHIV, inflammatory damage may have been cumulative given the delayed initiation of ART: the median age at ART initiation in our cohort was 6 years.

GDF-15 was more likely to be elevated in children with PHIV, including those with LV diastolic dysfunction and hypertrophy. The release of GDF-15 increases in response to cardiomyocyte tissue injury such as in the context of LV hypertrophy and dilated cardiomyopathy [33]. In a group of elderly individuals, GDF-15 was associated with lower ejection fraction, concentric LV remodelling and hypertrophy. Of note, elevated GDF-15 level was associated with interventricular septum and LV posterior wall z-scores, a finding previously reported among hypertensive patients with LV hypertrophy [34]. The findings in the present study suggest that elevated GDF-15 in children with PHIV may be an important indicator of myocardial remodelling. Several reports have shown an association of GDF-15 with all-cause mortality, subclinical cardiovascular disease, endothelial dysfunction, and LV hypertrophy independent of conventional risk factors. This suggests that GDF-15 may provide additional information on the risk of cardiovascular disease over and above traditional risk factors [35].

Although the level of ST2 was significantly higher in children with PHIV, it was not associated with any cardiac abnormalities. In contrast, Secemsky *et al* found that ST2 was associated with LV diastolic dysfunction among adults with HIV [14]. More recently, elevated ST2 levels were associated with LV diastolic dysfunction among adults with HIV from Tanzania [36]. Our findings are different to published data in adults, possibly because the disease phenotypes may be at an earlier stage in children (but in time may develop into the adult phenotype). ST2 is a marker for myocardial fibrosis and several studies have reported an association with adverse cardiac outcomes and all-cause mortality [37–39].

ICAM-1 and VCAM-1 are markers of vascular injury. Impaired endothelial function promotes atherogenesis and hypertension, both of which are important risk factors for cardiac remodelling resulting in heart failure [40]. Both markers are associated with subclinical atherosclerosis and all-cause mortality in the general population [41] and are elevated in HIV infection [42]. Miller *et al* found that children with HIV had elevated ICAM-1 and VCAM-1 which was related to HIV disease severity (low CD4 counts and higher viral load) [43]. In this study, only VCAM-1 was elevated in children with PHIV and was associated with increased interventricular septum z-scores. VCAM-1 has previously been linked to LV wall thickness or LV mass indexes in patients with hypertension [44, 45]. This biomarker could potentially be used as an early marker for increased wall thickness in children with HIV. Unexpectedly, we found an associated decreased risk of LV diastolic dysfunction with every one unit increase of ICAM-1. This could be due to multiple testing.

It is notable that we reported a high prevalence (42%) of echocardiographic abnormalities in this cohort. There is evidence of a general burden of cardiac abnormalities in children with HIV from Sub-Saharan Africa and elsewhere, with estimates ranging between 14-89% [9, 10, 46–48]. Similarly, in a large contemporary cohort of adults with HIV, Mondy *et al* reported a high prevalence of subclinical cardiac abnormalities including LV diastolic dysfunction (26%), LV systolic dysfunction (18%) and pulmonary hypertension in 57% [49]. Other studies have also corroborated this high burden in adults with HIV in this ART era [50, 51].

The panel of biomarkers investigated in this study illustrate different processes in the development of HIV-related cardiomyopathy, including myocardial insult, inflammation, and cardiac remodelling [18]. GDF-15 and ST2 are emerging biomarkers, which may have the same potential as natriuretic peptides to impact the way cardiac disease is evaluated and managed [18]. We propose that measuring circulating biomarkers will be important in young people with HIV to enable early detection of cardiac complications and provide pathophysiological insights. This facilitates prompt interventions to reduce cardiovascular morbidity during the transition to adulthood. Furthermore, these biomarkers might be able to replace more expensive and difficult to access tests including echocardiography, particularly in resource limited settings, although further prospective studies are needed to confirm this. Our findings also provide evidence of a link between inflammatory biomarkers and PHIV comorbidities as previously reported [22].

The primary limitation of this study was its cross-sectional design. Serial measurements of biomarkers may have provided incremental prognostic evidence and reflected changes in myocardial remodelling over time. However, single-time measurements of biomarkers have been shown to be predictive of adverse cardiovascular outcomes [52]. This study had limited statistical power to model infrequent outcomes. We did not have data on metabolic parameters including lipids and insulin resistance, which are important risk factors for cardiovascular disease. Data on echocardiographic abnormalities in children without HIV was lacking and, therefore, it is unknown if these associations exist among children without HIV. Cardiac MRI data may have provided additional insights into myocardial inflammation. Longitudinal follow-up of children with PHIV, together with integration of metabolic data, will be necessary to understand whether our findings translate to cardiovascular events.

In conclusion, CRP, TNF-α, ST2, VCAM-1 and GDF-15 were elevated in children with PHIV and established on ART. CRP and GDF-15 were associated with echocardiographic abnormalities in PHIV and provide insights into possible role of inflammation in the comorbidities of children with PHIV.

## Figures and Tables

**Table 1:** Clinical characteristics of the participants with and without HIV

Characteristics	HIV+ N= 195 Mean (SD)	HIV- N=211 Mean (SD)	P-value
Female, n (%) ^*a*^	94 (48.0)	109 (52.0)	0.487
Age, y	10.7 (2.6)	10.8 (2.8)	0.671
Height-for-age z-score ^*b,*^	-1.27 (1.1)	-0.27 (1.1)	**<0.001**
Stunted, N (%) ^*a, c,*^	48 (25.0)	15 (7.0)	**<0.001**
Weight-for age z-score ^*b, d*^	-1.11 (1.3)	-0.23 (1.1)	**<0.001**
Wasted, N (%) ^*a, c, d*^	44 (23.0)	8 (4.0)	**<0.001**
Body surface area, m^2^	1.05 (0.2)	1.18 (0.3)	**<0.001**
**Viral load**			
Viral load, copies/ml, median (IQR)	19 (19 – 208)		
Viral suppression, (<400 copies/ml) n (%)	152 (78)		
CD4 count, cells/μl, median (IQR) ^*d*^	710 (473 – 899)		
CD4 count<200, (cells/mm^3^), N (%)	9 (5)		
Age at HIV diagnosis, y, median (IQR) ^*d*^	5 (3 – 7)		
Age at ART start, y, median (IQR) ^*d*^	6 (3 – 8)		
Duration on ART, y, median (IQR) ^*d*^	4.8 (2.7 – 6.4)		
**ART regimen**			
2 NRTI + PI, n (%)	24 (21)		
2 NRTI + NNRTI, n (%)	88 (75)		
Unknown, n (%)	5 (4)		

a *chi-squared test*

b *Weight-for-age and height-for-age z-scores were calculated using British 1990 Growth Reference [53].*

c *Z-score <-2.*

d *Missing values: Weight-for age z-score- n=1 HIV+, n=1 HIV-; Body surface area- n=2 HIV+, n=1 HIV-: CD4 count- n=3; Age at ART start- n=3; Age at HIV diagnosis- n=1; Duration on ART- n=2* *y, years; SD, standard deviation; IQR, interquartile range; BMI, body mass index; BP, blood pressure; ART, antiretroviral therapy; NRTI, nucleoside reverse transcriptase; PI, protease inhibitor; NNRTI, non-nucleoside reverse transcriptase.*

**Table 2:** Multiple Linear Regression of Biomarkers and Echocardiographic z-scores in the HIV group.

	Univariable		Multivariable	
	Univariate β ± SE	P-value	Adjusted β ± SE	P-value
**LVdd z-scores**				
*Log_10_ CRP*	0.179 ± 0.095	0.063	0.185± 0.095	0.053
*Log_10_ TNF-alpha*	0.150 ± 0.089	0.093	0.137 ± 0.096	0.154
*Log_10_ IL-6*	0.082 ± 0.112	0.460	0.169 ± 0.122	0.167
*Log_10_ ST2/IL-33R*	0.152 ± 0.094	0.108	0.162 ± 0.092	0.081
*Log_10_ VCAM-1*	0.090 ± 0.075	0.231	0.083 ± 0.076	0.279
*Log_10_ ICAM-1*	0.052 ± 0.062	0.409	0.058 ± 0.059	0.329
*Log_10_ GDF-15*	0.227 ± 0.106	**0.034**	0.174 ± 0.113	0.128
**IVSd z-scores**				
*Log_10_ CRP*	0.029 ± 0.07	0.680	0.021 ± 0.07	0.774
*Log_10_ TNF-alpha*	0.137 ± 0.079	0.086	0.145± 0.079	0.068
*Log_10_ IL-6*	0.032 ± 0.075	0.062	0.027 ± 0.081	0.740
*Log_10_ ST2/IL-33R*	0.025 ± 0.079	0.752	0.020 ± 0.08	0.805
*Log_10_ VCAM-1*	0.144 ± 0.079	0.069	0.177 ± 0.08	**0.029**
*Log_10_ ICAM-1*	0.098 ± 0.077	0.206	0.083± 0.077	0.283
*Log_10_ GDF-15*	0.288 ± 0.078	**<0.001**	0.317 ± 0.083	**<0.001**
**LVPWd z-scores**				
*Log_10_ CRP*	0.129 ± 0.078	0.098	0.142 ± 0.079	0.074
*Log_10_ TNF-alpha*	0.013 ± 0.822	0.872	0.051± 0.087	0.557
*Log_10_ IL-6*	0.171 ± 0.090	0.058	0.249 ± 0.097	**0.011**
*Log_10_ ST2/IL-33R*	0.075± 0.080	0.354	0.113 ± 0.085	0.185
*Log_10_ VCAM-1*	0.032 ± 0.072	0.657	0.089 ± 0.078	0.257
*Log_10_ ICAM-1*	0.088± 0.087	0.313	0.073 ± 0.088	0.404
*Log_10_ GDF-15*	0.242 ± 0.089	**0.008**	0.327 ± 0.100	**0.001**
**LA z-scores**				
*Log_10_ CRP*	0.0.191 ± 0.115	0.099	0.198 ± 0.109	0.073
*Log_10_ TNF-alpha*	0.154 ± 0.097	0.114	0.172 ± 0.105	0.102
*Log_10_ IL-6*	0.069 ± 0.153	0.653	0.136 ± 0.152	0.372
*Log_10_ ST2/IL-33R*	0.169 ± 0.101	0.099	0.196 ± 0.102	0.057
*Log_10_ VCAM-1*	0.045 ± 0.076	0.553	0.102 ± 0.087	0.247
*Log_10_ ICAM-1*	0.041 ± 0.069	0.550	0.009± 0.071	0.891
*Log_10_ GDF-15*	0.064 ± 0.126	0.611	-0.012 ± 0.136	0.928
**RVdd z-scores**				
*Log_10_ CRP*	-0.052 ± 0.115	0.652	-0.065 ± 0.106	0.541
*Log_10_ TNF-alpha*	-0.027 ± 0.106	0.793	-0.029 ± 0.094	0.753
*Log_10_ IL-6*	-0.030 ± 0.122	0.807	0.015 ± 0.118	0.899
*Log_10_ ST2/IL-33R*	-0.075 ± 0.097	0.442	-0.087 ± 0.098	0.377
*Log_10_ VCAM-1*	-0.029 ± 0.077	0.708	0.063 ± 0.081	0.438
*Log_10_ ICAM-1*	-0.013 ± 0.078	0.861	-0.092 ± 0.076	0.228
*Log_10_ GDF-15*	-0.028 ± 0.125	0.818	-0.250 ± 0.247	**0.037**
**TAPSE z-scores**				
*Log_10_ CRP*	0.022 ± 0.076	0.766	0.020 ± 0.076	0.794
*Log_10_ TNF-alpha*	-0.033 ± 0.075	0.654	-0.050 ± 0.082	0.544
*Log_10_ IL-6*	-0.045 ± 0.080	0.573	-0.051 ± 0.094	0.586
*Log_10_ ST2/IL-33R*	-0.105 ± 0.069	0.131	-0.119 ± 0.076	0.123
*Log_10_ VCAM-1*	0.012 ± 0.060	0.839	0.023 ± 0.067	0.730
*Log_10_ ICAM-1*	-0.066 ± 0.067	0.327	-0.077 ± 0.069	0.266
*Log_10_ GDF-15*	-0.006 ± 0.085	0.938	-0.049 ± 0.095	0.602

*Multivariable model adjusted for age, sex, and body surface area and log_10_ viral load. Each row of the table represents a separate linear regression model. P-values were not corrected for multiple testing.* *Abbreviations: SE, standard error; IL-6, interleukin 6, TNF-α, tumour necrosis factor-alpha; CRP, C-reactive protein; GDF-15, growth differentiating factor-15, ST2, suppression of tumorigenicity 2; ICAM-1, intercellular adhesion molecule 1; VCAM-1, vascular cell adhesion molecules 1.*

**Table 3:** Logistic Regression of Individual Biomarkers and cardiac abnormalities in the HIV group

	Any cardiac abnormality		LV diastolic dysfunction		LV Hypertrophy		LA dilatation		RV dilatation	
	Adjusted Odds Ratio		Adjusted Odds Ratio		Adjusted Odds Ratio		Adjusted Odds Ratio		Adjusted Odds Ratio	
	aOR (95% CI)	P-value	aOR (95% CI)	P-value	aOR (95% CI)	P-value	aOR (95% CI)	P-value	aOR (95% CI)	P-value
Log_10_ CRP	1.50 (1.10 – 2.03)	**0.010**	1.49 (1.02 – 2.18)	**0.040**	1.31 0.82 – 2.09)	0.253	1.16 (0.68 – 1.96)	0.585	1.16 (0.66- 2.06)	0.605
Log_10_ TNF-alpha	0.94 (0.69 – 1.30)	0.729	0.84 (0.60 – 1.18)	0.324	0.88 (0.55 – 1.40)	0.578	1.12 (0.59 – 2.17)	0.715	1.17 (0.83- 1.64)	0.379
Log_10_ IL-6	1.16 (0.84 – 1.61)	0.364	1.08 (0.75 – 1.55)	0.674	1.28 (0.78 – 2.10)	0.335	1.33 (0.79 – 2.27)	0.285	0.87 (0.19-3.98)	0.859
Log_10_ ST2/IL-33R	1.35 (0.99 – 1.85)	0.061	1.28 (0.88 – 1.84)	0.195	1.21 (0.75 – 1.93)	0.438	0.92 (0.52 – 1.62)	0.764	1.18 (0.74- 1.89)	0.479
Log_10_ VCAM-1	1.18 (0.89 – 1.57)	0.243	1.13 (0.84– 1.53)	0.424	1.20 (0.81 – 1.78)	0.374	0.92 (0.53 – 1.58)	0.753	1.01 (0.71- 1.44)	0.947
Log_10_ ICAM-1	0.86 (0.66 – 1.11)	0.248	0.73 (0.56 – 0.96)	**0.023**	0.99 (0.60 – 1.66)	0.987	1.13 (0.57 – 2.22)	0.725	1.03 (0.53- 1.99)	0.939
Log_10_ GDF-15	1.53 (1.03 – 2.27)	**0.035**	1.71 (1.17 – 2.52)	**0.006**	1.84 (1.10 – 3.10)	**0.021**	1.03 (0.42 – 2.54)	0.942	0.88 (0.36- 2.13)	0.774

*Models were adjusted for age-category, sex, body surface area and viral suppression. Odds ratios represent the odds of having an abnormality for a one-unit change in biomarker level with one-unit representing one standard deviation*
